# Circulating *N*-lactoyl-amino acids and *N*-formyl-methionine reflect mitochondrial dysfunction and predict mortality in septic shock

**DOI:** 10.1007/s11306-024-02089-z

**Published:** 2024-03-06

**Authors:** Robert S. Rogers, Rohit Sharma, Hardik B. Shah, Owen S. Skinner, Xiaoyan A. Guo, Apekshya Panda, Rahul Gupta, Timothy J. Durham, Kelsey B. Shaughnessy, Jared R. Mayers, Kathryn A. Hibbert, Rebecca M. Baron, B. Taylor Thompson, Vamsi K. Mootha

**Affiliations:** 1 Department of Molecular Biology, Massachusetts General Hospital, Boston, MA USA; 2 Broad Institute, Cambridge, MA USA; 3 Division of Pulmonary and Critical Care, Massachusetts General Hospital, Boston, MA USA; 4 Division of Pulmonary and Critical Care, Brigham & Women’s Hospital, Boston, MA USA; 5 Department of Systems Biology, Harvard Medical School, Boston, MA USA; 6 Howard Hughes Medical Institute, Boston, MA USA

**Keywords:** Sepsis, Septic shock, Lactoyl, Lac-Phe, f-Met, Kynurenine

## Abstract

**Introduction:**

Sepsis is a highly morbid condition characterized by multi-organ dysfunction resulting from dysregulated inflammation in response to acute infection. Mitochondrial dysfunction may contribute to sepsis pathogenesis, but quantifying mitochondrial dysfunction remains challenging.

**Objective:**

To assess the extent to which circulating markers of mitochondrial dysfunction are increased in septic shock, and their relationship to severity and mortality.

**Methods:**

We performed both full-scan and targeted (known markers of genetic mitochondrial disease) metabolomics on plasma to determine markers of mitochondrial dysfunction which distinguish subjects with septic shock (n = 42) from cardiogenic shock without infection (n = 19), bacteremia without sepsis (n = 18), and ambulatory controls (n = 19) – the latter three being conditions in which mitochondrial function, proxied by peripheral oxygen consumption, is presumed intact.

**Results:**

Nine metabolites were significantly increased in septic shock compared to all three comparator groups. This list includes *N*-formyl-l-methionine (f-Met), a marker of dysregulated mitochondrial protein translation, and *N*-lactoyl-phenylalanine (lac-Phe), representative of the *N*-lactoyl-amino acids (lac-AAs), which are elevated in plasma of patients with monogenic mitochondrial disease. Compared to lactate, the clinical biomarker used to define septic shock, there was greater separation between survivors and non-survivors of septic shock for both f-Met and the lac-AAs measured within 24 h of ICU admission. Additionally, tryptophan was the one metabolite significantly decreased in septic shock compared to all other groups, while its breakdown product kynurenate was one of the 9 significantly increased.

**Conclusion:**

Future studies which validate the measurement of lac-AAs and f-Met in conjunction with lactate could define a sepsis subtype characterized by mitochondrial dysfunction.

**Supplementary Information:**

The online version contains supplementary material available at 10.1007/s11306-024-02089-z.

## Introduction

Sepsis is a syndrome characterized by organ dysfunction caused by dysregulated inflammation in response to an acute infection (Singer et al., 2016). Sepsis imposes a high burden of morbidity and mortality, affecting 1.7 million US adult patients and contributing to 250,000 US adult deaths annually (Rhee et al., 2017). Patients with septic shock, defined as those who require vasopressor medications to maintain a mean arterial pressure (MAP) > 65 mmHg and have a lactate > 2 mmol/L in the absence of hypovolemia, have a ~ 40% in-hospital mortality rate (Singer et al., 2016). Despite widespread adoption of clinical protocols to promptly address infection and provide critical organ support, sepsis incidence and mortality remains stubbornly high (Rhee & Klompas, 2020), and no therapies are available that directly regulate the cellular metabolic derangements that contribute to organ failure.

The syndromic definition of sepsis is necessarily broad to facilitate prompt recognition and treatment by clinicians, yet sepsis encompasses marked heterogeneity based on the diversity of underlying patient characteristics, pathogens and immune responses (Leligdowicz & Matthay, 2019, Prescott et al., 2016). Thus, a major focus of current sepsis research is to define sepsis subtypes which might be amenable to specific therapies (Maslove et al., 2022). Significant progress has been made in defining sepsis subtypes (Sinha et al., 2023) based on blood transcriptomics (Scicluna et al., 2017, Wong et al., 2011), monocyte expression levels of human leukocyte antigens (Bodinier et al., 2021) and aggregating demographic, vital signs and clinical laboratory data (Seymour et al., 2019, Shankar-Hari et al., 2021). Still, a major challenge remains in relating these phenotypes to specific cellular perturbations that can be therapeutically targeted (Maslove et al., 2022).

A candidate molecular subtype of sepsis is mitochondrial dysfunction (Singer, 2014, Arulkumaran et al., 2016). Mitochondrial dysfunction is a prominent feature of experimental sepsis models and includes ultrastructural distortion (Wang et al., 2014), decreased enzymatic activity (Piel et al., 2008) and gene expression (Callahan & Supinski, 2005) of components of the electron transport chain (ETC), inhibition of TCA cycle enzymes (Vary, 1996) and impaired mitochondrial biogenesis (Tran et al., 2011, Mannam et al., 2014). Mitochondrial dysfunction has been proposed as a mechanism underlying a unique physiological state often associated with septic shock in which oxygen consumption is not augmented by increased oxygen delivery (Ronco et al., 1993) [“cytopathic dysoxia”(Fink, 1997)], but this could also be explained by maldistribution of circulation and microcirculatory dysfunction resulting in true tissue hypoxia (Rovas et al., 2019, Sakr et al., 2004). Given the difficulties in obtaining tissue from vital organs from septic patients, it remains challenging to determine the extent to which mitochondrial dysfunction is a causal factor in human septic organ dysfunction. Studies assessing mitochondrial function in humans with sepsis have reached differing conclusions depending on the tissues studied and the assays used. For example, in skeletal and cardiac muscle and in platelets, significant quantitative reductions in mitochondrial content (Fredriksson et al., 2006), gene expression (Matkovich et al., 2017) and ETC enzymatic activity (Protti et al., 2015, Carre et al., 2010) have been reported, and upregulation in skeletal muscle of PGC1-alpha, the master regulator of mitochondrial biogenesis, is highly correlated with sepsis survival and recovery (Carre et al., 2010). By contrast, studies of peripheral blood immune cells in sepsis have reported increased mitochondrial content and respiration, and no correlation between these parameters and survival were observed (Sjovall et al., 2013). Because there is no clinically available gold standard by which to define ETC dysfunction acutely, circulating biomarkers of mitochondrial dysfunction are needed. Towards the goal of better defining ETC dysfunction in sepsis, in this study we employed a metabolomics platform enriched with a panel of metabolites discovered and validated by our laboratory that are known to be increased in genetic mitochondrial disease (Sharma et al., 2021), in which there is known ETC dysfunction.

We collected venous blood from a cohort of 98 subjects at a single academic medical center and compared those with septic shock to those with cardiogenic shock without infection, bacteremia without sepsis, and ambulatory controls—three conditions in which peripheral oxygen extraction, as a measure of mitochondrial function, is intact (Boekstegers et al., 1991, Boekstegers et al., 1994). We chose these comparator groups of acutely ill patients to highlight metabolic derangements specific to the unique physiological state of septic shock. We analyzed differential abundance of 176 metabolites in each sample using two platforms: (1) full-scan metabolomics which quantified the relative abundance of 159 metabolites of known molecular identity and (2) targeted absolute quantitation of an additional 17 metabolites recently identified by our laboratory as being markers of genetic mitochondrial disease (Sharma et al., 2021). Included in our targeted measurements is a recently described family of metabolites, *N*-lactoyl-amino acids (lac-AAs), reported here for the first time in acutely ill subjects. Lac-AAs were first described in 2015 as metabolites present throughout mammalian tissues, formed by the reverse proteolysis of lactate and amino acids, and catalyzed by the ubiquitous protease cytosolic nonspecific dipeptidase 2 (CNDP2) (Jansen et al., 2015). We previously identified plasma resting levels of four lac-AAs—lac-leucine/isoleucine (lac-Leu/Ile), lac-phenylalanine (lac-Phe), lac-tyrosine (lac-Tyr), lac-valine (lac-Val)—as amongst the very best circulating biomarkers of MELAS (Mitochondrial Encephalopathy Lactic Acidosis Stroke-like episodes) (Sharma et al., 2021), the most prevalent monogenic disease caused by mutations in mitochondrial DNA (mtDNA).

In the current work, we identified a unique subset of metabolites which is significantly differentially abundant in septic shock in comparison to all other groups. Our findings demonstrate that: (1) *N*-formyl-methionine (f-Met), a known marker of dysregulated mitochondrial protein translation (Cai et al., 2021), is one of 9 metabolites significantly increased in septic shock compared to all other groups; (2) lac-Phe is significantly increased in septic shock compared to all other groups; and (3) compared to lactate, the clinical biomarker used to define septic shock, there is greater separation between survivors and non-survivors of septic shock for both f-Met and the lac-AAs measured within 24 h of ICU admission. In addition, our metabolomics dataset yielded novel findings of metabolites uniquely increased or decreased in septic shock which will stimulate additional research to understand their prognostic and therapeutic implications.

## Results

### Cohort description and clinical characteristics

We enrolled 98 subjects: 42 with septic shock, 19 with cardiogenic shock with no clinical evidence of infection, 18 with bacteremia stable on a medical floor without hypotension or elevated lactate, and 19 ambulatory controls (Table 1). The age distribution was similar across groups; three of the four groups had more males, while the bacteremia without sepsis group was majority female. Key clinical parameters of hematologic, renal, and hepatic function are provided in Table 1 and are consistent with the greater illness severity of the septic shock group. The septic shock group had a significantly higher Sequential Organ Failure Assessment (SOFA) score than the cardiogenic shock group (7.1 ± 2.8 vs. 4.0 ± 3.6, *P* < 0.001). The central venous oxygen saturation of septic shock subjects was significantly higher than the mixed venous oxygen saturation of cardiogenic shock subjects (78.1 ± 9.2 vs. 63.1 ± 10.1, *P* < 0.001; central venous oxygen saturations were rarely obtained by the clinical team caring for cardiogenic shock subjects who had pulmonary artery catheters). Fourteen of 42 (33%) septic shock subjects died in the hospital. Dosages of vasopressors and inotropic medications, use of mechanical circulatory support, site(s) of infection and microbiology data are provided in Supplemental Table 1. Of the 42 septic shock subjects, 24 had pneumonia, 10 had urinary tract infection, 9 had intra-abdominal infection including *Clostridium difficile* colitis, 7 had skin and soft tissue infection and 3 had primary bloodstream infection from endocarditis or intravenous drug use. Twenty-nine septic shock subjects (69%) had positive microbial data with a diverse set of common pathogens including gram negative rods, methicillin-susceptible and methicillin-resistant *Staphylococcus aureus*, *Streptococcus pneumoniae* and Influenza A. Eleven septic shock subjects (26%) had positive blood cultures.

**Table 1:** Demographic and Clinical Characteristics

	Ambulatory controls	Bacteremia without sepsis	Cardiogenic shock	Septic shock
n	19	18	19	42
Age, mean (SD)	62.8 (17.1)	59.2 (14.6)	61.3 (15.2)	65.3 (16.6)
% Female	47.4%	61.1%^#^	26.3%	33.3%
Hemoglobin, g/dL, mean (SD)	13.5 (2.3)***	9.4 (1.7)	11.8 (2.3)	10.6 (2.7)
Leukocytes, cells × 10^9/L mean (SD)	7.3 (2.4)***	10.1 (6.8)*	10.2 (5.0)*	15.6 (8.3)
Platelets, cells × 10^3/uL mean (SD)	258 (97)*	181 (110)	173 (83)	178 (88)
Creatinine, mg/dL mean (SD)	1.1 (0.3)*	1.1 (0.7)*	1.7 (1.1)	2.4 (2.2)
Total Bilirubin, mg/dL mean (SD)	0.5 (0.2)	0.9 (0.8)	1.1 (0.6)	1.7 (2.2)
SOFA exclusive of GCS, mean (SD)	N/A	N/A	4.0 (3.6)***	7.1 (2.8)
Venous oxygen saturation, %, mean (SD)	N/A	N/A	63.1 (10.1)***	78.1 (9.2)

Statistical significance assessed relative to septic shock group

No significant group differences (P < 0.05) with respect to age or sex

^*^ = P < 0.05, ** = P < 0.01, *** = P < 0.001, **** = P < 0.0001

^#^Not significant, P = 0.09

### Identification of metabolites increased or decreased in septic shock

**Fig. 1:**
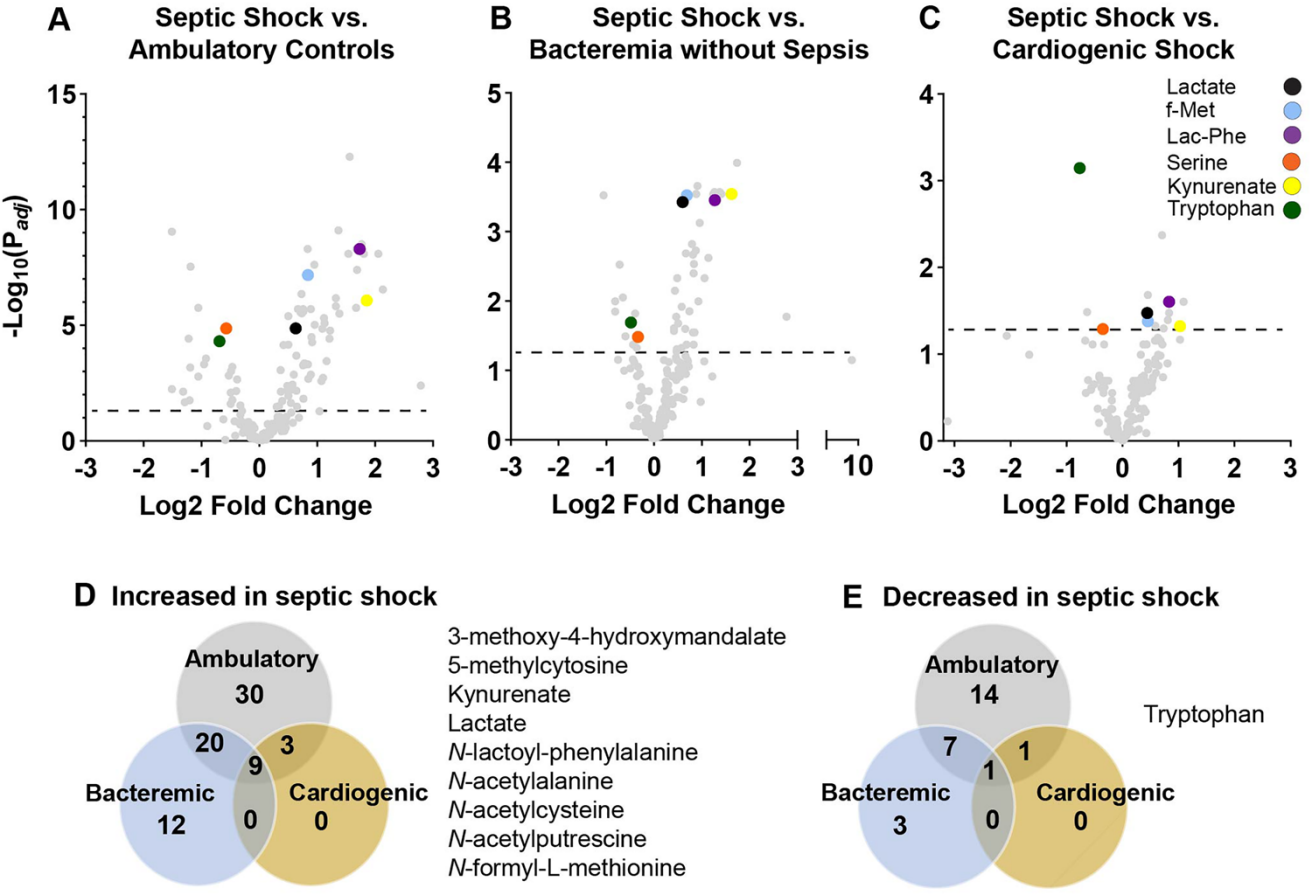
Metabolomics of septic shock in comparison to ambulatory controls, bacteremia without sepsis and cardiogenic shock adjusted for age and sex. Volcano plots of septic shock (n = 42) in comparison to **A** ambulatory controls (n = 19), **B** bacteremia without sepsis (n = 18), and **C** cardiogenic shock (n = 19). Dashed lines denote P_*adj*_ = 0.05. Venn diagrams quantifying the metabolites significantly (P_*adj*_ < 0.05) **D** increased and **E** decreased in comparison to septic shock with an accompanying list of the metabolites significantly different in septic shock in comparison to all three comparator groups. “f-Met” = *N*-formyl-l-methionine; “Lac-Phe” = *N*-Lactoyl-phenylalanine

We compared the levels of 176 metabolites (see Methods) in septic shock in comparison to ambulatory controls (Fig. 1A, Supplemental Table 2), bacteremia without sepsis (Fig. 1B, Supplemental Table 3) and cardiogenic shock (Fig. 1C, Supplemental Table 4). Adjusted for age and sex and using a false discovery rate of 0.05, in septic shock 35% of metabolites were significantly increased and 13% were significantly decreased compared to ambulatory controls, 23% were significantly increased and 6% were significantly decreased compared to bacteremia without sepsis, and 7% were significantly increased and 1% were significantly decreased compared to cardiogenic shock. Though our dataset does not include historical clinical laboratory values, plasma creatinine levels were higher in the shock groups, potentially indicative of greater renal dysfunction, and decreased renal clearance could contribute to differences in metabolite abundance between groups. Our findings were robust to the inclusion of creatinine as a covariate (Supplemental Fig. 1; Supplemental Tables 5–7).

We identified 9 metabolites that were significantly increased (P_*adj*_ < 0.05) in septic shock compared to all other groups (Fig. 1D). Tryptophan was the only metabolite (P_*adj*_ < 0.05) that was significantly decreased in septic shock compared to all other groups (Fig. 1E). This list of metabolites highlights several key metabolic derangements in septic shock. First, both lactate and 3-methoxy-4-hydroxymandelate, a breakdown product of catecholamines (Eisenhofer et al., 2004), were significantly increased in septic shock in comparison to all other groups. Second, tryptophan was decreased and its breakdown product kynurenate was increased, consistent with increased tryptophan catabolism through the kynurenine pathway (Zeden et al., 2010). Third, the anti-oxidant N-acetylcysteine (NAC), which has been studied as a therapy in sepsis clinical trials (Szakmany et al., 2012), was significantly increased. Our findings that 5-methylcytosine, N-acetylalanine and N-acetylputrescine are significantly increased in septic shock have not been explored in detail previously.

### Focused analysis of f-Met and lac-AAs—metabolites significantly increased in septic shock compared to all other groups

**Fig. 2:**
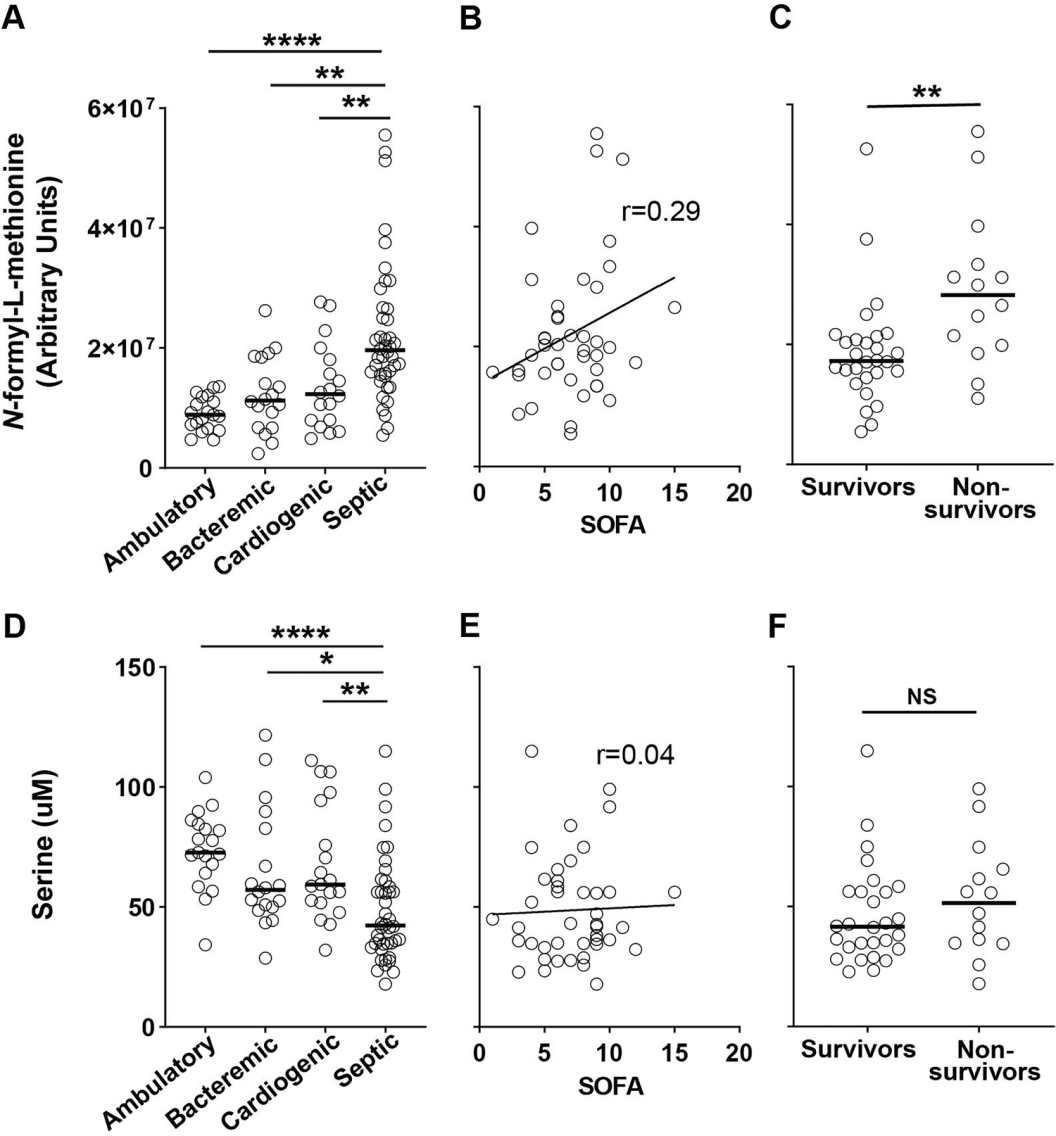
Relationship of f-Met and serine to sepsis severity and mortality. **A** Relative quantification of f-Met for all subjects. Solid line denotes median. Kruskal–Wallis multiple comparisons test of statistical significance. **B** Correlation of f-Met and Sequential Organ Failure Assessment (SOFA) score for septic shock subjects. Pearson correlation coefficient. **C** Relative quantification of f-Met in septic shock 28-day survivors (n = 28) vs. non-survivors (n = 14). Mann–Whitney test of statistical significance. **D** Absolute quantitation of serine for all subjects. Solid line denotes median. Kruskal–Wallis multiple comparisons test of statistical significance. **E** Correlation of serine and SOFA score for septic shock subjects. Pearson correlation coefficient. **F** Absolute quantitation of serine in septic shock 28-day survivors (n = 28) vs. non-survivors (n = 14). Mann–Whitney test of statistical significance. * = P < 0.05, ** = P < 0.01, *** = P < 0.001, **** = P < 0.0001

Elevated circulating f-Met is a marker of disrupted mitochondrial protein translation linked to overall increased prevalence of common age-associated diseases and all-cause mortality in the general population (Cai et al., 2021). In sepsis, circulating *N*-formyl peptides including f-Met contribute to sepsis pathophysiology through damage-associated molecular pattern (DAMP) signaling which amplifies tissue-damaging inflammation (Harrington et al., 2017) and inhibits neutrophil defense against secondary nosocomial infection (Kwon et al., 2021). As has been described recently in two large ICU cohorts (Sigurdsson et al., 2022), we found that f-Met is not only increased in septic shock (Fig. 2A), but also trended towards being positively correlated with sepsis severity (ρ = 0.29, P = 0.07; Fig. 2B) and was significantly increased in septic shock 28-day non-survivors vs. survivors (P < 0.01; Fig. 2C). We observed both increased f-Met and decreased serine (Fig. 2D, Supplemental Table 2–4) in our septic shock subjects. Serine was significantly decreased in septic shock in comparison to ambulatory controls (Log2 Fold Change = − 0.57, P_*adj*_ < 0.0001) and bacteremia without sepsis (Log2 Fold Change = − 0.34, P_*adj*_ = 0.03), and decreased at the level of nominal significance in comparison to cardiogenic shock (Log2 Fold Change = − 0.35, P = 0.005, P_*adj*_ = 0.051). Serine is a key intermediary in both the cytosolic and mitochondrial portions of one-carbon metabolism which leads to the production of 10-formyl-tetrahydrofolate, the source of the formyl group in f-Met. In contrast to f-Met, we did not observe a relationship between serine and sepsis severity (Fig. 2E) or mortality (Fig. 2F).

**Fig. 3:**
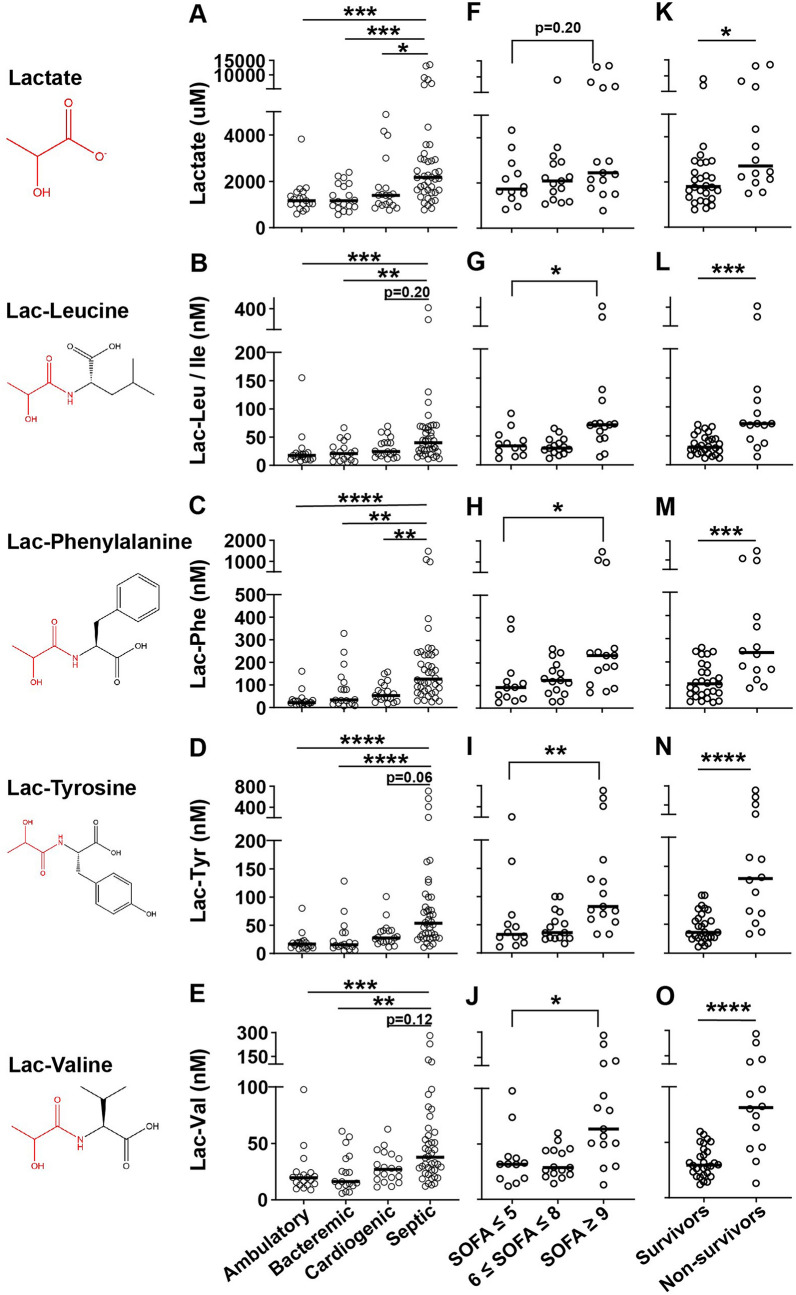
*N*-Lactoyl-amino acids vs. lactate as distinguishers of sepsis severity and mortality. Absolute quantitation of all subjects (**A**–**E**); septic shock subjects divided by SOFA score (n = 12, 15, 15) (**F**–**J**); and septic shock 28-day survivors (n = 28) vs. non-survivors (n = 14) (**K**–**O**) for the indicated metabolite. For comparisons of illness groups and septic shock SOFA groups, Kruskal–Wallis multiple comparisons test of statistical significance. For comparisons of septic shock mortality, Mann–Whitney test of statistical significance. * = P < 0.05, ** = P < 0.01, *** = P < 0.001, **** = P < 0.0001

Given our recent identification of lac-Phe as amongst the metabolites most significantly elevated in genetic mitochondrial disease and most positively correlated with disease severity and heteroplasmy (Sharma et al., 2021), we found it notable that lac-Phe was amongst the 9 metabolites increased in septic shock compared to all other groups. We therefore analyzed the levels of lactate and each of the 4 lac-AAs in our dataset, and compared how each varied between groups, and within the septic shock group as a function of severity (split by SOFA tertile) and mortality. Both lactate and the lac-AAs were most elevated in the septic shock group (Figs. 3A–E). Lactate trended towards being increased in the highest SOFA tertile compared to the lowest (mean: 4592 uM vs. 2098 uM, Kruskal–Wallis P = 0.20; Fig. 3F). All of the lac-AAs were statistically significantly increased in the highest as compared to the lowest SOFA tertile (lac-Leu/Ile highest SOFA tertile mean: 103.3 nM vs. lowest SOFA tertile mean: 37.7 nM, Kruskal–Wallis P = 0.01; lac-Phe: 376.1 nM vs. 126.5 nM, P = 0.02; lac-Tyr: 180.0 nM vs. 58.5 nM, P < 0.01; lac-Val: 90.1 nM vs. 36.7 nM, P = 0.01; Figs. 3G–J). Lactate was significantly increased in non-survivors compared to survivors (mean: 4749 uM vs. 2347 uM, Mann–Whitney P = 0.01, Fig. 3K). Compared to lactate, there was greater separation between non-survivors and survivors for each of the lac-AAs (lac-Leu/Ile non-survivors mean: 108.2 nM vs. survivors mean: 34.9 nM, Mann–Whitney P < 0.001; lac-Phe: 418.6 nM vs. 118.1 nM, P < 0.001; lac-Tyr: 203.6 nM vs. 45.6 nM, P < 0.0001; lac-Val: 98.5 nM vs. 32.5 nM, P < 0.0001; Figs. 3L–O). Moreover, whereas 29% of survivors had lactate less than the lowest value in non-survivors, 43% of survivors had lac-Phe less than the lowest value in non-survivors; 14% of non-survivors had lactate greater than the highest value in survivors, while 43% of non-survivors had lac-Phe greater than the highest value in survivors. When considering future potential use of lac-AAs as clinical biomarkers, it is noteworthy that the levels of these four lac-AAs are very highly correlated with one another (ρ for the correlation of log values between 0.81 and 0.96, Supplemental Fig. 2A–L), allowing for substantial information on the group to be obtained from measurement of just one.

**Fig. 4:**
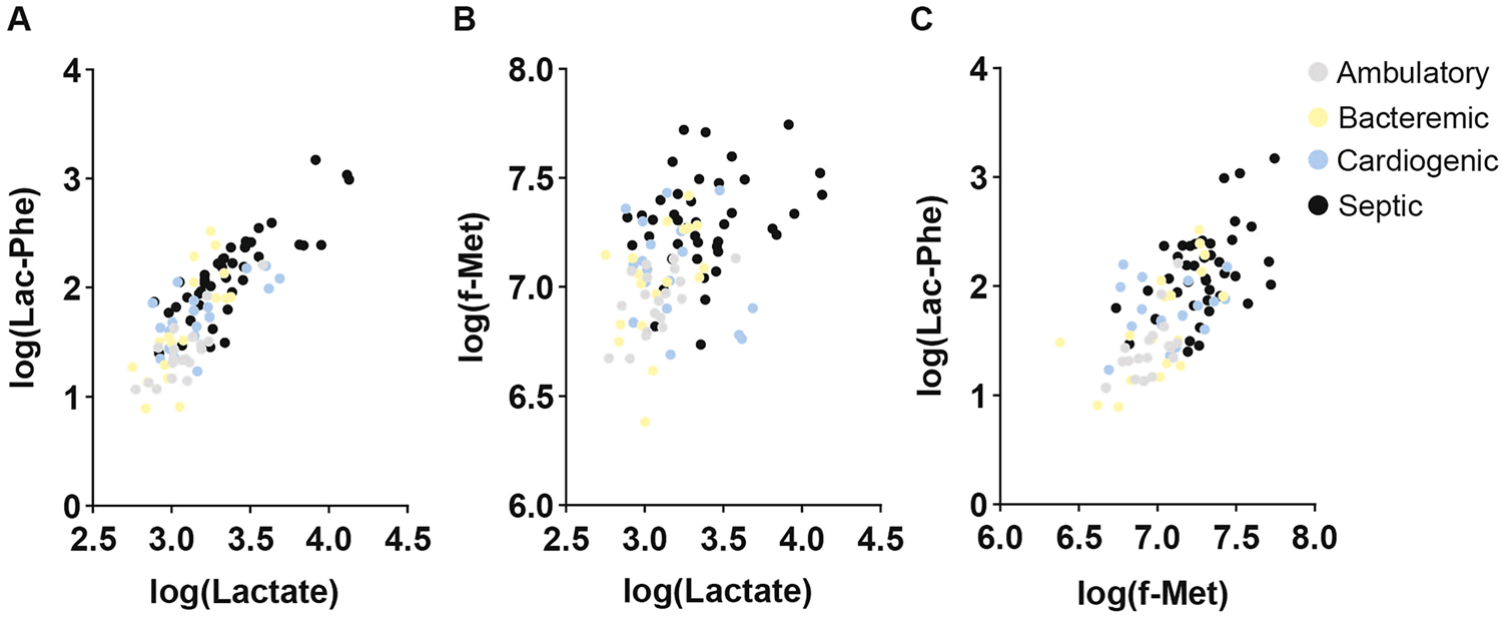
Interrelationship of lactate, f-Met and Lac-Phe. Correlation of **A** log (lac-Phe) and log (lactate); **B** log (f-Met) and log (lactate); and **C** log (lac-Phe) and log (f-Met) for all subjects color-coded by group

Finally, we assessed the extent of conditional independence of lactate, f-Met, and lac-Phe to provide insight on the additional informational value of measuring both f-Met and lac-Phe in a septic patient. The strong positive correlation between lactate and the lac-AAs is maintained across all groups (Supplemental Fig. 2M–T; Fig. 4A). Interestingly, while lactate and f-Met were significantly positively correlated in ambulatory controls (ρ = 0.56, P < 0.05; Fig. 4B) and bacteremia without sepsis (ρ = 0.47, P < 0.05), in the critical illness groups they were not significantly correlated (cardiogenic shock: ρ = − 0.32, P = 0.20; septic shock ρ = 0.28, P = 0.07). Similarly, lac-Phe and f-Met were positively correlated in septic shock (ρ = 0.47, P < 0.01; Fig. 4C) though to a lesser extent than in ambulatory controls (ρ = 0.64, P < 0.01), suggesting that septic shock pathophysiology perturbs both lac-Phe and f-Met through mechanisms that are not entirely overlapping.

## Discussion

To our knowledge, this is the first study to compare the metabolome of septic shock to that of both bacteremia without sepsis and cardiogenic shock. This novel comparison highlighted that both f-Met and the lac-AAs measured within the first 24 h of ICU admission achieved greater separation between septic shock survivors and non-survivors than lactate, the clinical biomarker currently most widely used to define septic shock based on consensus definitions. [An elevated lactate > 2 mmol/L was included in the Sepsis-3 definition of septic shock because when combined with fluid-refractory hypotension, it increases the identification of patients at risk for in-hospital mortality (~ 42% vs. ~ 30% for those with lactate ≤ 2 mmol/L) (Singer et al., 2016).] At present, there is no clinically available gold standard by which to define acute ETC dysfunction in hospitalized patients; the techniques which support the diagnosis of genetic mitochondrial disease, such as muscle biopsy with histochemical analysis (Parikh et al., 2015), are impractical to deploy at scale in the critically ill and would not necessarily reflect the status of vital organs. Because high central venous oxygen saturation—which could reflect any combination of impaired oxygen extraction from ETC dysfunction, supranormal cardiac output or peripheral shunting from microcirculatory dysfunction (Haase & Perner, 2011)—is associated with increased mortality in septic shock (Textoris et al., 2011), a circulating biomarker which distinguishes these distinct physiologic states would help to define different subtypes of sepsis physiology. However, no such circulating biomarker has yet been defined. As the field currently lacks such gold standard tests to identify ETC dysfunction, in this study we sought to provide insight—though certainly not a definitive answer—into the question of the presence of ETC dysfunction in sepsis by measuring metabolites which are known markers of genetic mitochondrial disease. Our findings that metabolite markers of genetic mitochondrial disease are increased in septic shock and positively associated with severity and mortality provides suggestive evidence that ETC dysfunction is present in at least a subset of septic shock subjects, opening the door for further studies to validate biomarkers of ETC dysfunction in septic shock.

Our results are concordant with prior studies of metabolic perturbations in sepsis. A recent comprehensive review of 26 metabolomics studies comparing patients with sepsis with either healthy controls or patients with sterile inflammation highlighted that metabolic pathways involving cellular bioenergetics are amongst the most consistently perturbed in sepsis (Hussain et al., 2022). Consistent with the broader literature comparing sepsis to healthy controls, we too found increases in metabolites reflective of impaired energy production, including lactate, pyruvate, the lactate-to-pyruvate ratio, alpha-hydroxybutyrate and beta-hydroxybutyrate (Supplemental Table 2).

Strikingly, we found that tryptophan was the one metabolite significantly (P_*adj*_ < 0.05) decreased in septic shock in comparison to all other groups. Indeed, we found that in addition to tryptophan being significantly decreased in septic shock in comparison to all other groups, kynurenine was significantly increased in comparison to ambulatory controls (Supplemental Table 2) and bacteremia without sepsis (Supplemental Table 3) while trending towards being increased in comparison to cardiogenic shock (Supplemental Table 4), and kynurenine’s direct breakdown product kynurenate was one of the 9 metabolites significantly increased in septic shock in comparison to all other groups. Tryptophan is broken down to kynurenine by the enzyme indoleamine 2,3-dioxygenase (IDO), the expression of which is significantly increased by the inflammatory cytokine interferon-γ (Darcy et al., 2011). Increased tryptophan catabolism through the kynurenine pathway, marked by an increased kynurenine to tryptophan ratio (Darcy et al., 2011), is a predictor of the development of sepsis and increased illness severity in a variety of clinical contexts, including trauma (Zeden et al., 2010), community-acquired pneumonia (Meier et al., 2017) and COVID-19 (Lionetto et al., 2021). Moreover, increased IDO expression in the vasculature during septic shock is correlated with the severity of hypotension (Changsirivathanathamrong et al., 2011). Our study extends the observations from this literature that the extent of tryptophan catabolism via the kynurenine pathway is increased in the highly inflammatory state of septic shock even compared to other acute illness states.

Our findings generate important questions to be addressed in future work about whether and through what mechanisms lac-AAs and f-Met might have a causal effect on sepsis pathophysiology. The lac-AAs exert potent biological effects in vivo. Anorexia is a major conserved feature of the host response to infection (van Niekerk et al., 2016) and lac-Phe contributes significantly and directly to the curtailment of appetite immediately post-exercise (Li et al., 2022). It is intriguing to speculate whether lac-AAs might have a direct toxic effect in sepsis. Animal studies to test this hypothesis are feasible. While little is known about the role of CNDP2 (the enzyme essential for lac-AA production) in normal physiology, CNDP2 KO mice were recently generated and found to have increased susceptibility to hepatic and renal injury from acetaminophen overdose because of insufficient generation of the antioxidant glutathione (Kobayashi et al., 2021). Experimental sepsis studies in CNDP2 KO mice could provide mechanistic insights into the function of lac-AAs.

Consistent with studies in three large ICU cohorts (Kwon et al., 2021, Sigurdsson et al., 2022) and consistent with studies of ICU molecular epidemiology that circulating levels of mitochondrial constituents (mtDNA (Johansson et al., 2018, Wang et al., 2020) and *N*-formyl-peptides (Kwon et al., 2021)) are associated with deleterious ICU outcomes, we found that f-Met is increased in septic shock and positively correlated with both severity and mortality. It has been generally assumed that the increased circulating f-Met in sepsis reflects mitochondrial dysfunction, but because f-Met is also produced by bacteria, its origin is not certain. To our knowledge, this is the first study to compare plasma f-Met levels in septic shock subjects (only one-quarter of whom were clinically bacteremic) with subjects with frank bacteremia. Our finding that f-Met was significantly increased in septic shock relative to bacteremia without sepsis provides support for the host mitochondrial as opposed to bacterial origination of f-Met. If indeed circulating f-Met is mitochondrial in origin, the process by which it enters the plasma under pathophysiological conditions is an important research question, with possibilities including: (1) that excess accumulation of f-Met in the mitochondria results in transport to the cytosol and then to the extracellular space or that (2) the enzyme MTFMT which loads the formyl group onto methionyl-tRNA and is normally restricted to the mitochondria engages cytosolic methionyl-tRNA under conditions of stress (Lee et al., 2022). Whether increased circulating levels of f-Met are effectors or merely markers of mitochondrial dysfunction is an active area of investigation, but a potential causal role is supported by the finding that supplementing fibroblast cell media with exogenous f-Met decreases mitochondrial protein translation, levels of ETC subunits and cellular respiration, and triggers the integrated stress response (Cai et al., 2021). Animal studies are required to resolve both the mechanism and effect of increased plasma f-Met in sepsis.

To our knowledge, this is the first study to report that plasma levels of serine are decreased in septic shock in comparison to other acute illness states. This is consistent with a recent report that serine is decreased in states of human insulin resistance (Fridman et al., 2021) (and septic shock is characterized by marked insulin resistance) (NICE-SUGAR Study Investigators, 2009). In mice, serine deficiency is sufficient to induce peripheral neuropathy through perturbations in de novo sphingolipid biosynthesis (Handzlik et al., 2023). It is therefore reasonable to speculate on whether decreased serine availability—perhaps driven by consumption of serine in one-carbon metabolism to support nucleotide synthesis for rapidly proliferating immune cells (Pearce et al., 2013)—is causally related to the peripheral neuromyopathy of critical illness, a major driver of post-ICU morbidity (Vanhorebeek et al., 2020). Clinical trials of serine supplementation for rare congenital peripheral neuropathies have shown it to be safe and likely efficacious (Fridman et al., 2019). Our finding provides a rationale for preclinical studies to explore the effect of serine supplementation in supporting peripheral nerve health in sepsis.

We acknowledge several important limitations of this study. First, our cohort of 98 subjects represents a convenience sample at a single tertiary care facility. Though the early ICU illness severity and 28-day mortality in our septic shock subjects were similar to those of large cohorts enrolled in recent prominent ICU clinical trials, our findings on the predictive value of lac-AAs must be replicated in a larger independent cohort before they can be the basis of further biomarker development. Second, for each subject we collected samples at a single time point, and so we are unable to report on metabolite trajectories, or how a metabolite’s relationship to organ dysfunction and mortality evolves over the course of an ICU admission. Third, as is true of all observational studies of ICU patients, there is significant heterogeneity not only in demographics and clinical comorbidities, but also in duration of illness before medical presentation, recent nutrition and initial therapies delivered prior to sample collection that we are unable to explicitly control for in our analyses. Fourth, while we believe that our results distinguishing septic shock from cardiogenic shock are driven by the distinct pathophysiology of each condition, the cardiogenic shock subjects had less organ failure and lower lactate levels than the septic shock subjects. This was a consequence of the fact that all subjects were enrolled within 24 h of ICU admission, and those cardiogenic shock subjects who clinically deteriorate often do so very rapidly after admission, often before their healthcare agents could be approached about study participation. Fifth, a limitation of the strength of our inference about the mitochondrial as opposed to bacterial origin of f-Met is that non-septic bacteremic subjects were enrolled after blood cultures resulted positive, by which time antibiotic therapy usually sterilized the blood collected for our study.

Nevertheless, the employment of a unique choice of comparator groups and the absolute quantitation of the lac-AAs in acutely ill hospitalized patients are novel features of this study and have yielded new insights into the metabolomic readout of mitochondrial dysfunction in septic shock. Because they are related to core perturbations in host metabolism during sepsis, lac-AAs, f-Met, and serine warrant further study to determine their effector functions in critical illness and their potential utility in defining sepsis subtypes.

### Methods

All subjects (or legal surrogates) provided informed consent to participate in this study, which was approved by the Mass General Brigham IRB (Protocol 2017P002436). A convenience sample was assembled comprising adults between the ages of 21 and 90 who were recruited from a single academic medical center between March 2018 and April 2019 and met criteria for septic shock, cardiogenic shock, bacteremia without sepsis or ambulatory controls as defined in detail in the Extended Methods. Survivors were defined as those surviving at least 28 days from ICU admission or to hospital discharge. Clinical characteristics of the 134 subjects with MELAS, MELAS carriers and controls for whom data is shown in Supplemental Fig. 2 has been reported previously (Sharma et al., 2021).

Clinical laboratory values are reported from the time closest to and before 8 am on the day of sample collection. Reported SOFA scores were calculated at 8 am on the day of sample collection and ranged from 0 to a potential maximum of 20 as the Glascow Coma Scale was not assessed. Reported central and mixed venous oxygenation saturations are from the time closest to and before (within 24 h of) sample collection. Sample collection and processing are described in the Extended Methods.

Our metabolite profiling and quantification consisted of two workflows to quantify 176 metabolites considered in our analysis: (1) full-scan metabolomics yielding relative quantification of over 5000 peaks of which 159 have known molecular identity; and (2) focused absolute quantitation of the metabolites previously identified as biomarkers of MELAS (Sharma et al., 2021), 17 of which (4 lac-AAs, glucose, 6 acylcarnitine species, 3 β-hydroxy-carnitine species, 3 β-hydroxy-fatty acid species) were distinct from the 159 relatively quantified metabolites. Comprehensive details of our metabolomics method including the creation of calibration curves and pooled QC samples are described in the Extended Methods.

Data analysis was performed using Tracefinder™ 4.1 with 5 ppm mass tolerance and the quality of integration for each chromatographic peak was reviewed. For imputation of relatively quantified metabolites, zero values were converted to ½ the lowest non-zero value of that metabolite prior to analysis. Differential analysis of metabolites was performed using the empirical Bayesian method implemented by the R limma package (version 3.50.3), adjusted for age, sex and creatinine as indicated. The resulting P-values were adjusted with the Benjamini and Hochberg method for multiple hypothesis testing correction. For comparisons of f-Met, serine, lactate and the lac-AAs between study groups, statistical significance was calculated in Graphpad Prism (version 9.5) using the Kruskall–Wallis test for multiple comparisons or Mann–Whitney test for two-group comparisons. See Extended Methods for further details.

### Supplementary Information

Below is the link to the electronic supplementary material.

Supplementary file1 (JPG 209 KB)—Metabolomics of septic shock in comparison to ambulatory controls, bacteremia without sepsis and cardiogenic shock adjusted for age, sex and creatinine. Volcano plots of septic shock (n = 42) in comparison to **A** ambulatory controls (n = 19), **B** bacteremia without sepsis (n = 18), and **C** cardiogenic shock (n = 19). Dashed lines denote Padj = 0.05. Venn diagrams quantifying the metabolites significantly (Padj < 0.05) **D** increased and **E** decreased in comparison to septic shock with an accompanying list of the metabolites significantly different in septic shock in comparison to all three comparator groups. “f-Met” = *N*-formyl-l-methionine; “Lac-Phe” = *N*-Lactoyl-phenylalanine

Supplementary file2 (JPG 359 KB)—Correlations amongst *N*-lactoyl-amino acids and with lactate. Correlations of each of log (lac-Leu/Ile), log (lac-Phe), log (lac-Tyr), log (lac-Val) with one another amongst the 98 subjects in this study (**A**–**F**) and in MELAS patients, carriers and controls (n = 134) in a previously reported cohort (Sharma, 2021) (**G**–**L**). Correlations of the log of each of the above lac-AAs with log (lactate) in the current (**M**–**O**) and previously reported cohort (**Q**–**T**). Pearson correlation coefficients

Supplementary file3 (PDF 106 KB)

Supplementary file4 (XLSX 19 KB)

Supplementary file5 (XLSX 18 KB)

Supplementary file6 (XLSX 18 KB)

Supplementary file7 (XLSX 19 KB)

Supplementary file8 (XLSX 18 KB)

Supplementary file9 (XLSX 18 KB)

Supplementary file10 (DOCX 24 KB)

## Data Availability

All data and code associated with this study are available from the authors upon request. The data is available at the NIH Common Fund’s National Metabolomics Data Repository (NMDR), the Metabolomics Workbench, https://metabolomicsworkbench.org, where it has been assigned Project ID ROHITSHARMA_20240103_094523.
