# Supplementary figures and images for: Circulating N-lactoyl-amino acids and N-formyl-methionine reflect mitochondrial dysfunction and predict mortality in septic shock
Source: Metabolomics. 2024 Mar 6;20(2):36. doi: 10.1007/s11306-024-02089-z (PMC10917846; doi:10.1007/s11306-024-02089-z)

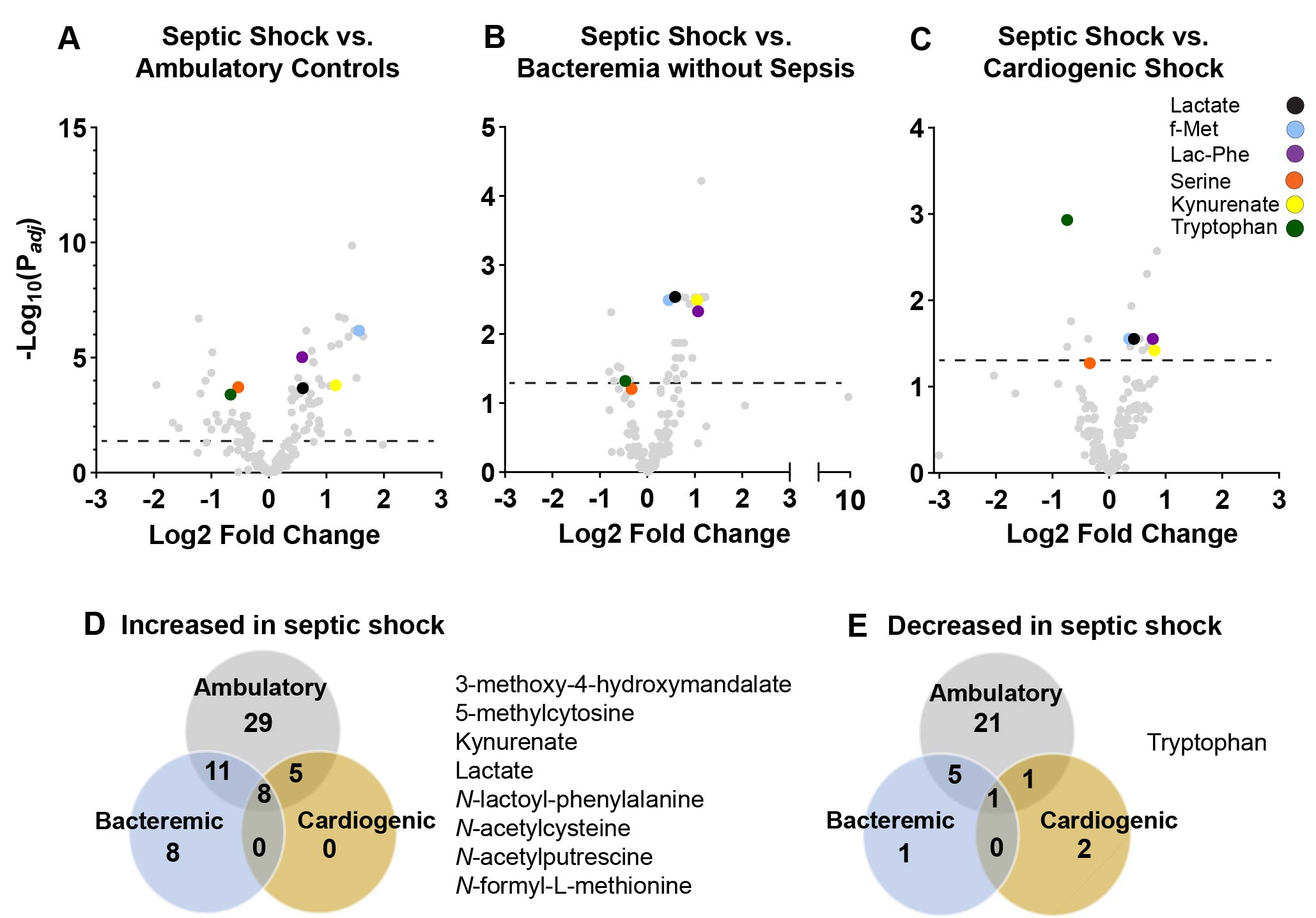

Supplement: Supplementary file 1 — Supplementary file1 (JPG 209 KB)—Metabolomics of septic shock in comparison to ambulatory controls, bacteremia without sepsis and cardiogenic shock adjusted for age, sex and creatinine. Volcano plots of septic shock (n = 42) in comparison to A ambulatory controls (n = 19), B bacteremia without sepsis (n = 18), and C cardiogenic shock (n = 19). Dashed lines denote Padj = 0.05. Venn diagrams quantifying the metabolites significantly (Padj < 0.05) D increased and E decreased in comparison to septic shock with an accompanying list of the metabolites significantly different in septic shock in comparison to all three comparator groups. “f-Met” = N-formyl-l-methionine; “Lac-Phe” = N-Lactoyl-phenylalanine [file 11306_2024_2089_MOESM1_ESM.jpg]

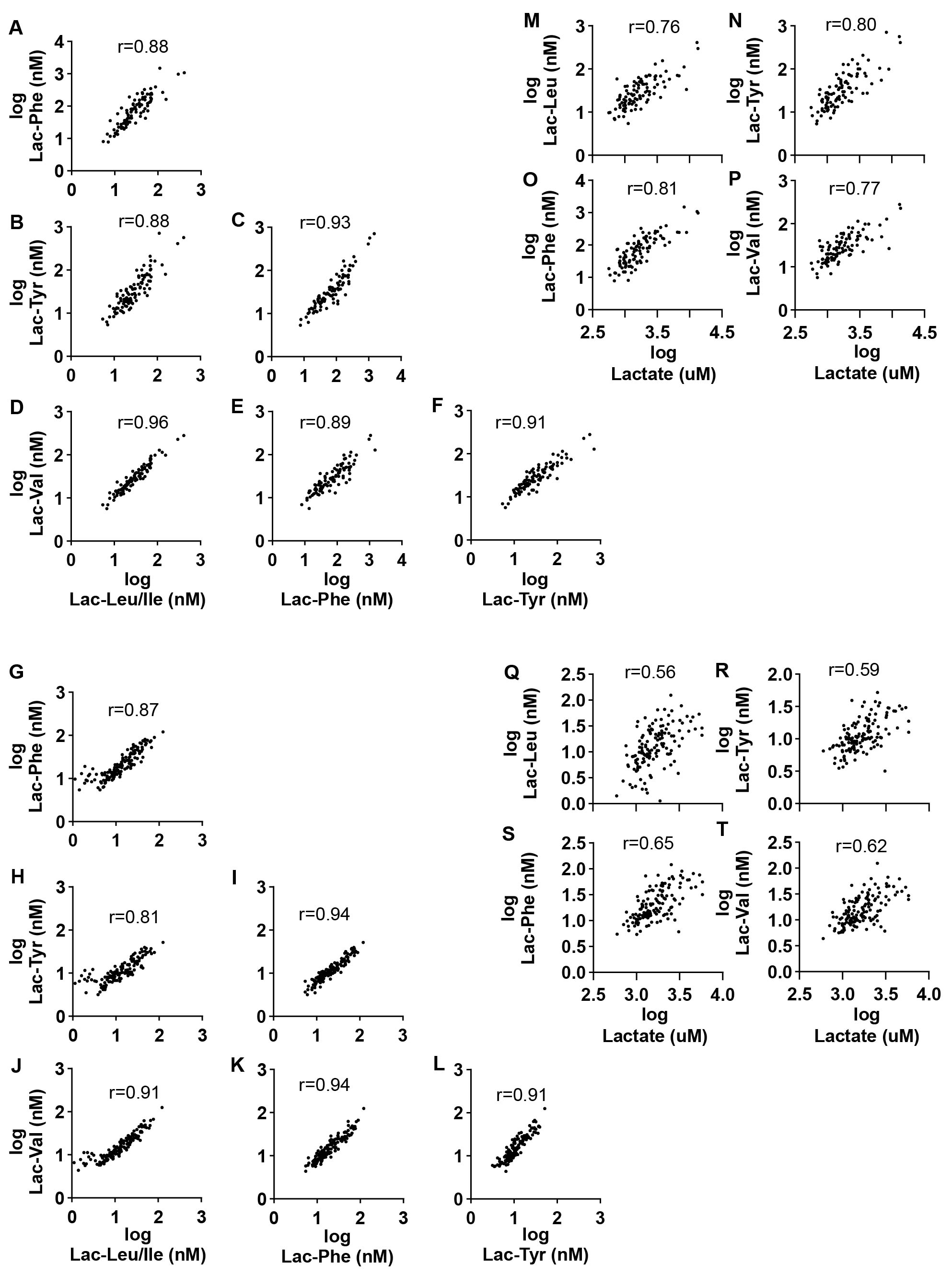

Supplement: Supplementary file 2 — Supplementary file2 (JPG 359 KB)—Correlations amongst N-lactoyl-amino acids and with lactate. Correlations of each of log (lac-Leu/Ile), log (lac-Phe), log (lac-Tyr), log (lac-Val) with one another amongst the 98 subjects in this study (A–F) and in MELAS patients, carriers and controls (n = 134) in a previously reported cohort (Sharma, 2021) (G–L). Correlations of the log of each of the above lac-AAs with log (lactate) in the current (M–O) and previously reported cohort (Q–T). Pearson correlation coefficients [file 11306_2024_2089_MOESM2_ESM.jpg]
