# Supplementary material for: Circulating N-lactoyl-amino acids and N-formyl-methionine reflect mitochondrial dysfunction and predict mortality in septic shock
Source: Metabolomics. 2024 Mar 6;20(2):36. doi: 10.1007/s11306-024-02089-z (PMC10917846; doi:10.1007/s11306-024-02089-z)
Supplement: Supplementary file 3 — Supplementary file3 (PDF 106 KB) [file 11306_2024_2089_MOESM3_ESM.pdf]

Supplemental Table 1. Extended Clinical Information

**Cardiogenic Shock Subjects (n=19)**	
Left Ventricular Ejection Fraction, mean (SD)	24 (15)
% receiving inotropic support	95
% receiving mechanical circulatory support	28
**Septic Shock Subjects (n=42)**	
Norepinephrine-equivalent vasopressor dose, mean (SD)	25.6 (26.1)
Source of Infection, %*:	
Pneumonia	57
Urinary Tract	24
Intra-abdominal	21
Skin & Soft Tissue	17
Direct Bloodstream & Endocarditis	7
Organisms, n:	
Clostridium difficile	3
Coagulase-negative *Staphylococcus* species	3
Corneybacterium species	2
Escherichia coli	4
Enterococcus species	4
Haemophilus influenzae	1
Influenza A	3
Klebsiella species	6
Methicillin-resistant *Staphylococcus aureus*	4
Methicillin-sensitive *Staphylococcus aureus*	2
Pseudomonas aeruginosa	1
Serratia marcescens	1
Streptococcus agalactiae	1
Streptococcus pneumoniae	2
Unspeciated gram negative rods	2
No positive microbiological data	13

*total exceeds 100% as subjects could have multiple sources of infection
