# Supplementary material for: Circulating N-lactoyl-amino acids and N-formyl-methionine reflect mitochondrial dysfunction and predict mortality in septic shock
Source: Metabolomics. 2024 Mar 6;20(2):36. doi: 10.1007/s11306-024-02089-z (PMC10917846; doi:10.1007/s11306-024-02089-z)
Supplement: Supplementary file 10 — Supplementary file10 (DOCX 24 KB) [file 11306_2024_2089_MOESM10_ESM.docx]

**Extended Methods**

*Subjects*

All subjects (or legal surrogates) provided informed consent to participate in this study, which was approved by the Mass General Brigham IRB (Protocol 2017P002436). A convenience sample was assembled comprising adults between the ages of 21 and 90 who were recruited from a single academic medical center between March 2018 and April 2019 according to the following criteria:

Septic shock – admitted to an ICU within the past 24 hours with a source of infection, receiving antibiotics and vasopressors to maintain a mean arterial blood pressure > 65 mmHg and with lactate > 2 mg/dL upon presentation;

Cardiogenic shock – admitted to a cardiac ICU within the past 24 hours with a left ventricular ejection fraction < 40%, receiving inotropic medications and/or mechanical circulatory support with no documented source of infection and not receiving antibiotics (except for prophylaxis against commensal skin flora if mechanical circulatory support was used);

Bacteremia without sepsis – admitted to a medical floor with a blood culture positive for a pathogenic organism within the past 24 hours and never having had lactate > 2 mg/dl, systolic blood pressure < 90 mmHg or having received vasopressors during this admission;

Ambulatory controls – ambulatory patients presenting for a Level 1 cardiopulmonary exercise test for evaluation of dyspnea with left ventricular ejection fraction ≥ 55%, normal arterial oxygen saturation and resting lactate on ambient air and no history or subsequent diagnosis of mitochondrial disease.

Exclusion criteria were pregnancy, enrollment in hospice and any use of extracorporeal membrane oxygenation.

Clinical characteristics of the 134 subjects with MELAS, MELAS carriers and controls for whom data is shown about the correlation amongst lac-AAs and between lactate and the lac-AAs in Supplemental Figure 2 has been reported previously (Sharma, 2021).

*Clinical data*

Basic clinical laboratory values (complete blood cell count and comprehensive metabolic panel) are reported from the time closest to and before 8 am on the day of sample collection. Reported SOFA scores were calculated at 8 am on the day of sample collection and ranged from 0 to a potential maximum of 20 as the Glascow Coma Scale was not assessed. Reported central and mixed venous oxygenation saturations are from the time closest to and before (within 24 hours of) sample collection.

*Sample collection and processing*

10 mL of whole venous blood was collected in an EDTA-containing tube, placed on ice and promptly centrifuged at 4°C at 1500g for 15 minutes. Plasma was divided into 250ul to 500ul aliquots and stored at -80°C until analysis.

*Plasma metabolite profiling and quantification*

Our metabolite profiling and quantification consisted of two workflows to quantify 176 metabolites considered in our analysis: 1) full-scan metabolomics yielding relative quantification of over 5000 peaks of which 159 have known molecular identity; and 2) focused absolute quantitation of the metabolites previously identified as biomarkers of MELAS (Sharma, 2021), 17 of which (4 lac-AAs, glucose, 6 acylcarnitine species, 3 β-hydroxy-carnitine species, 3 β-hydroxy-fatty acid species) were distinct from the 159 relatively quantified metabolites. We measured lactate and serine using both methods (they were tightly correlated). The relative quantification values were included for generation of volcano plots (Figure 1, Supplemental Figure 1), whereas absolutely quantitated values were used for the subsequent figures in which these metabolites were analyzed in further detail.

Comprehensive details of our metabolomics method have been reported previously (Sharma, 2021). In brief, 30 µl of plasma sample was mixed with 137 µl of ice-cold acetonitrile containing internal standards (^13^C_6_ -glucose, ^13^C_3_-lactate, ^13^C_3_-pyruvate, D3-α-HB,^13^C_2_ -β-HB, ^13^C_3_ -alanine,^13^C_3_-serine, ^13^C_2_-glycine, D9-carnitine, D3-creatine, D6-succinate, Cambridge isotope-labeled carnitine standard B) for metabolite extraction. Samples were vortexed and incubated on ice for 30 minutes. After centrifugation for 20 minutes at 4°C and 21,000g, 90 µl of the sample was transferred to an autosampler glass vial for LCMS analysis and 10 µl of the sample was injected on Waters XBridge amide column (2.1x100 mm, 2.5µm, Part # 186006091). The pooled QC sample was prepared by mixing equal volumes of each sample and injected every few samples to evaluate the analytical performance. Samples were injected in a randomized order to avoid any run order effect.

Calibration curves for the absolute concentration measurements were generated in a surrogate matrix buffer. 4% w/v human serum albumin in PBS was used as a surrogate matrix buffer. Calibration curves were prepared from 38.4 µM to 10.2 mM for lactate, 0.4 µM to 401.6 µM for pyruvate, 0.24 µM to 31.0 µM for α-ketobutyrate, 10 µM to 1145.2 µM for acetoacetate, 6.4 µM to 420.7 µM for α-hydroxybutyrate, 4.0 µM to 1072.0 µM for β-hydroxybutyrate, 0.005 to 5.1 µM for C4:0 carnitine, 0.005 to 5.2 µM for C3:0-carnitine, 0.06 µM to 124.8 µM for C2:0-carnitine, 31.8 µM to 34.0 mM for glucose, 0.06 µM to 1049.7 µM for creatine, 2 µM to 1995.1 µM for alanine, 1.6 µM to 212.3 µM for carnitine, 32.7 µM to 1027.9 µM for glycine, 3.4 µM to 54.9 µM for succinate, 6.4 µM to 397.4 µM for serine, and 0.8 µM to 55.0 µM for malate. D3-α-HB was used as an internal standard for the acetoacetate. The column oven temperature was 27°C and the autosampler was 4C°. Mobile phase A was 5/95 acetonitrile/water, 20mM ammonium acetate, pH9 (adjusted with ammonium hydroxide) and mobile phase B was acetonitrile. The LC gradient conditions at flow rate of 0.220 mL/min were: 0 min 85%B, 0.5 min 85%B, 9 min 35%B, 11 min 2%B, 12min 2%B, 13.5 min 85%B, 14.6 min 85%B , 15 min 85%B with 0.420 mL/min to 18 min. Dionex Ultimate 3000 UHPLC system was coupled to Q-Exactive Plus Orbitrap mass spectrometer (Thermo Fisher Scientific, Waltham, MA) with HESI probe operating in switch polarity mode. MS parameters were: sheath gas flow 50, aux gas flow 10, sweep gas flow 2, spray voltage 2.50KV in negative & 3.8KV in positive, Capillary temperature 310°C, S-lens RF level -50 and aux gas heater temperature 370°C. Data acquisition was done using Xcalibur (Thermo Scientific) in range of: 70-1000 m/z, resolution 70,000, AGC target -3E6 and maximum injection time of 80 ms. Data analysis for the absolute concentration was performed using Tracefinder^TM^ 4.1 with 5 ppm mass tolerance and the quality of integration for each chromatographic peak was reviewed manually. Metabolite profiling analysis was performed with Compound Discoverer (version 3.2) using a cubic spline function with a max corrected QC area of RSD-30% to compensate for time-dependent batch effect. MS/MS and retention time or retention time of each metabolite were matched against a reference standard to confirm the identities.

Lactoyl-amino acids (lac-phenylalanine, lac-valine, lac-tyrosine, lac-leucine/isoleucine), β-hydroxy fatty acids (3-OH-C8:0, 3-OH-C10:0, 3-OH-C12:0) and acylcarnitine species (3-OH-C12:0-carnitine, 3-OH-C14:0-carnitine, 3-OH-C16:0-carnitine, C14:0 carnitine, C16:0 Carnitine, C5:0 carnitine) were quantified as described previously (Sharma, 2021). We were not able to distinguish the constitutional isomers lac-Leu and lac-Ile because they have the same retention times on our LC-MS method and their fragmentation patterns were also identical, which is also a feature of other platforms measuring lac-AAs (Jansen, 2015). In brief, 50µL of plasma or calibration standard was extracted with 200µL of methanol containing the internal standards (^13^C_3-_lac-V, ^13^C_3_-lac-Leu, ^13^C_3_-lac-Phe, ^13^C_3_-lac-Tyr, D3-OH-C16:0 carnitine, D3-C4:0 carnitine, 3-OH-C8:0-D12, Cambridge isotope-labeled carnitine standard B). Samples were vortexed and incubated on ice for 20 minutes. After centrifugation for 20 minutes at 4°C and 21,000g, 200 µl of the sample was dried down using the Genevac EZ-2 elite vacuum dryer. The dried extract was resuspended in 90 µL of 50/50 methanol/water, vortex, sonicated for 2 minutes in ice cold water bath, incubated on ice and centrifuge at 21,000g and 4°C for 20 minutes. The calibration curves were prepared with the above-mentioned surrogate matrix buffer and followed the same steps as the plasma sample extraction. The calibration curve range was 4.68 nM-1200 nM for lac-Val, 3.125 nM-400 nM for lac-Tyr and lac-Leu/Ile, 3.125 nM-800 nM for lac-Phe, 7.81 nM-2000 nM for C5:0 carnitine, 0.78nM-100 nM for C16:0-Carnitine and C14:0-Carnitine, 19.53 nM-5000 nM for 3-OH-C8:0, 0.781 nM-200 nM for 3-OH-C16:0 carnitine, 0.488 nM-1000 nM for 3-OH-C14:0 carnitine, 0.06 1nM-1000 nM for 3-OH-C12:0 carnitine, 1.95 nM-2000 nM for 3-OH-C12:0, 0.61 nM-10000 nM for 3-OH-C10:0. 4 µL of each sample was injected onto a Chromolith Performance RP-18e column (2.0 x 100 mm, Millipore).

*Data analysis and quality control*

Data acquisition was done using the same methodology described previously (Sharma, 2021). Data analysis was performed using Tracefinder^TM^ 4.1 with 5 ppm mass tolerance and the quality of integration for each chromatographic peak was reviewed. For imputation of relatively quantified metabolites, zero values were converted to ½ the lowest non-zero value of that metabolite prior to analysis. For full-scan data, column sums were carefully examined for each subject, and one subject (cardiogenic shock group) was a low outlier and thus omitted from downstream analyses of relatively quantified metabolites.

*Statistics*

Differential analysis of metabolites was performed using the empirical Bayesian method implemented by the R limma package (version 3.50.3) adjusted for age, sex (and creatinine for sensitivity analysis reported in Supplemental Figure 1 and Supplemental Tables 5-7). The resulting P-values were adjusted with the Benjamini and Hochberg method for multiple hypothesis testing correction. For comparisons of f-Met, serine, lactate and the lac-AAs between study groups, statistical significance was calculated in Graphpad Prism (version 9.5) using the Kruskall-Wallis test for multiple comparisons or Mann-Whitney test for two-group comparisons.
